# Supplementary material for: Influence of scar age, laser type and laser treatment intervals on adult burn scars: A systematic review and meta-analysis
Source: PLoS One. 2023 Sep 27;18(9):e0292097. doi: 10.1371/journal.pone.0292097 (PMC10529539; doi:10.1371/journal.pone.0292097)
Supplement: Attachment — Submitted filename: Reviewer comments.docx [file pone.0292097.s003.docx]

Overall, the text is well-written and presents a comprehensive meta-analysis of laser therapy for hypertrophic burn scars. The suggested changes aim to improve clarity, consistency, and readability throughout the text.

Abstract:

Line 37: Change "(-0.39 [95%CI= -0.68; -0.10], p=0.01)" to "(-0.39 [95%CI= -0.68; -0.10], p=0.01)" to correct the p-value.

Introduction:

Line 54: Consider rephrasing "patients with hypertrophic scarring struggle" to "patients with hypertrophic scarring experience" for clarity.

Line 58: Change "the impact on the body" to "the impact on the body's function" for better specificity.

Line 68: Add a space after "lower wave" for consistency.

Line 69: Consider rephrasing "are playing an increasingly important role" to "play an increasingly important role" for a more active voice.

Methods:

Line 118: Change "chosen to be excluded" to "were chosen for exclusion" for better readability.

Line 125: Add a hyphen between "ablation" and "therapy" to read "ablation-therapy" for consistency with other terms.

Line 136: Consider rephrasing "Articles in Chinese that were included in the title and abstract screening were translated to English" to "Articles written in Chinese were translated into English for inclusion in the title and abstract screening" for better clarity.

Results:

Line 242: Consider rephrasing "that tested five different outcomes" to "that investigated five different outcomes" for better clarity.

Line 241-245: Consider merging the sentences into two or three sentences to improve readability.

Discussion:

Line 332: Change "latent (>12 months since injury)" to "latent (>12 months since injury) scar therapy" for clarity.

Line 342: Consider rephrasing "PDLs work on the premise of targeting haemoglobin, resulting in selective photothermolysis of blood vessels, and are known to be safer than ablative lasers but less effective due to less penetration of skin" to "PDLs work by targeting hemoglobin in blood vessels, resulting in selective photothermolysis, and they are generally considered safer than ablative lasers but have less penetration depth" for better readability.

Line 372: Change "interval and laser type subgroup analyses, some of the results were based around a single study" to "interval and laser type subgroup analyses had limited data, with some of the results based on a single study" for clarity.

Conclusion:

Line 379: Consider rephrasing "Laser therapy is an effective method of management for hypertrophic burns scars with early or latent initiation" to "Laser therapy is an effective method of management for hypertrophic burn scars, with either early or latent initiation" for better readability.
